# Proteomic Mediators of Overall Cardiovascular Health on All-Cause Mortality

**DOI:** 10.3390/nu15030781

**Published:** 2023-02-03

**Authors:** Toshiko Tanaka, Sameera A. Talegawkar, Yichen Jin, Julián Candia, Giovanna Fantoni, Stefania Bandinelli, Luigi Ferrucci

**Affiliations:** 1 Longitudinal Studies Section, Translational Gerontology Branch, National Institute on Aging, Baltimore, MD 21224, USA; 2 Department of Exercise and Nutrition Sciences, Milken Institute School of Public Health, The George Washington University, Washington, DC 20052, USA; 3 Geriatric Unit, Azienda Sanitaria Firenze (ASF), 50137 Florence, Italy

**Keywords:** cardiovascular health, proteomics, mediation, all-cause mortality

## Abstract

Measures of cardiovascular health (CVH) assessed by a combination of behavioral and biological factors has shown protective associations with all-cause mortality. The mechanisms underlying these associations have not been fully elucidated. In this study, we characterized the plasma proteomics profile of CVH and tested whether specific proteins mediated the associations between CVH and all-cause mortality in participants of the InCHIANTI study. Of the 1301 proteins tested, 92 proteins were associated with CVH (22 positively, 70 negatively). Proteins most strongly associated with CVH included leptin (LEP), fatty acid binding protein 3 (FABP3), Angiopoietin-2 (ANGPT2), and growth-differential factor 15 (GDF15). Of the 92 CVH-associated proteins, 33 proteins significantly mediated the associations between CVH and all-cause mortality, with percent mediation ranging from 5 to 30%. The most significant mediating proteins were GDF15 and insulin-like growth factor 2 (IGFBP2). Proteins associated with better CVH were enriched for proteins that reflect the suppression of the complement coagulation and GH/IGF pathways.

## 1. Introduction

In 2010, the American Heart Association put forth a set of guidelines, called the Life’s Simple 7 (LS7), with a goal of improving cardiovascular health and reducing deaths from cardiovascular diseases and stroke by 20% in a decade. The LS7 encompasses seven components, including health factors (measures of body mass index, fasting blood glucose, total cholesterol, and blood pressure levels) and health behaviors (measures of diet quality, physical activity levels, and exposure to cigarette smoking). In developing the LS7, sets of criteria were defined for each of the health factors and behaviors based on evidence that they are associated with health benefits [1].

Since the establishment of the LS7, studies have developed scoring algorithms that classify and categorize study subjects based on achieving these established goals [2]. Better cardiovascular health (CVH), as assessed by the LS7, has shown protective associations with all-cause and cardiovascular mortality [3,4,5], incidence of cardiovascular events [2,6], cancer risk [2], aging-related declines in physical [7,8] and cognitive function [9,10,11], frailty [12], and psychosocial health and depression [13,14,15]. Taken together, these findings suggest that CVH captures biological age, and this notion is also supported by CVH being associated with the epigenetic clock [16,17], one of the widely studied molecular biomarkers of biological age.

The use of omics in epidemiological research has become widespread, aimed at identifying biomarkers of disease risk, its pathology, and progression. In populations studies, assessment of biomarkers is frequently performed in blood (plasma, serum) due to the accessibility of samples, as well as for the potential to translate findings of circulating biomarkers for clinical use. Different type of biological molecules can be measured in blood, including genetics, epigenetics, proteins, and metabolites. Each molecular layer provides insight into the biology and function of an individual. Proteomics is a particularly attractive omics tool for biomarker discovery, as proteins are the functional component of the molecular cascade and interpretation of proteomic results can be intuitive. Until recently, there were technical challenges for conducting proteomic studies due to the dynamic range of proteins in blood. However, recent developments in proteomic technology, such as the aptamer-based protein assessment method, has allowed for the simultaneous assessment of large number of circulating proteins [18]. These technological advancements have been a catalyst for the growing number of proteomic biomarker discovery studies for different health conditions. However, there have been no investigations that have focused on the proteomic signatures of CVH, examined using the Life’s Simple 7 metric, and whether specific biomarkers mediate the associations with adverse outcomes. To address this gap in knowledge, we characterized the proteomic profile of CVH in the InCHIANTI Study and investigated whether these proteins mediated the associations between overall CVH and mortality in the cohort participants.

## 2. Materials and Methods

### 2.1. Study Population

The InCHIANTI study is a population-based cohort study of aging in an older population recruited from the Chianti region in Tuscany, Italy. Detailed description aims and study design was published previously [19]. In brief, 1616 residents were selected as candidate study participants of the population registry of Greve in Chianti (a total of 11,709 residents with 19.3% of the population greater than 65 years of age), and Bagno a Ripoli (a total of 4704 residents, 20.3% of which were greater than 65 years of age). From these selected residents, 1453 participants (participation rate was 90%) were enrolled at baseline, conducted between 1998–2000. The distribution of participant ages ranged between 21–102 years. For this study, we used data from 703 subjects 65 years and older with complete data on plasma proteins and CVH. A structured interview by study staff was conducted to collected basic sociodemographic characteristics such as age and sex. A 24-h urine sample was collected at baseline to measure urinary creatinine clearance. Additionally, a 24-h urinary creatinine excretion that was <10 mg/kg of lean body mass in men and <8 mg/kg of lean body mass in women was considered inadequate. Data on mortality up to 20 years following the start of the study were evaluated through general registry. Mortality registry was based on the death certificates that are submitted immediately after the death of residents to the registry office of the municipality. The study protocol was approved by the Italian National Institute of Research and the Care of Aging Institutional Review and review board at the National Institutes of Health (Baltimore, MD, USA).

### 2.2. Cardiovascular Health

Cardiovascular health was assessed using LS7 criteria specified by the American Heart Association, which includes four health behaviors (smoking, physical activity, diet, and body mass index) and three heath factors (total cholesterol, fasting glucose, and blood pressure). Self-reported smoking status was collected during the structured interview and participants were categorized as smokers (within 3 years of the date of the interview), former smokers, or non-smokers. Participants reported their physical activity level from the past year (hardly any physical activity; mostly sitting/some walking; light exercise 2–4 h/week; moderate 1–2 h or light > 4 h/week; moderate exercise >3 h/week; intense exercise many times/week; walks 5+ km/day or 5+ days/week for 5+ years). Diet was assessed using a food frequency questionnaire. Overnight fasted blood samples were collected, and serum was aliquoted and stored at −80 °C. Total cholesterol was measured using enzymatic tests (Roche Diagnostics, Mannheim, Germany). Fasting glucose was measured with an automated system with an enzymatic colorimetric assay using the glucose-oxidase-peroxidase-chromogen reaction and a Roche analyzer (Roche Diagnostics, GmbH, Mannheim, Germany). Resting supine blood pressure was measured in both arms using a mercury sphygmomanometer three times. The average of the second and third measurements was used for both systolic and diastolic blood pressure. Each component was categorized into ideal, intermediate, and poor status.

Scoring criteria for smoking status, diet, and physical activity were modified considering data available for this cohort and to ensure better health promotion and translation. Smoking was coded as never smoked (ideal), former smoker (intermediate), and current smoker within 3 years (poor). Physical activity was coded as low (inactive, or with some walking, coded as poor), medium (light exercise 2–4 h/week, coded as intermediate), and high (light exercise for more than 4 h/week, moderate exercise for at least 1–2 h/week, or intense exercise many times/week, coded as ideal). Dietary intakes were assessed using a previously validated food frequency questionnaire that was originally developed for the European Prospective Investigation on Cancer and Nutrition study, and diet quality was assessed using the Mediterranean Diet Score (MDS), which evaluated the intake of six beneficial food groups and nutrients (vegetables, legumes, fruits and nuts, cereal, fish, and monounsaturated-to-saturated fatty acid ratio), two detrimental food groups (meat and dairy products), and alcohol, which was categorized based on sex-specific intake [20]. Participants received 1 point for intakes of beneficial food groups and nutrients greater than the sex-specific median, intakes of detrimental food groups less than the sex-specific median, or moderate alcohol intake, and received 0 points otherwise. The overall MDS was calculated by adding the score of each component. Diet quality was then categorized as ideal, intermediate, and poor based on overall MDS ranges 6–9, 4–5, and 0–3, respectively. Body mass index (BMI) was classified as normal weight (<25 kg/m^2^), overweight (25–29.9 kg/m^2^), and obese (≥30 kg/m^2^).

For health factors, total cholesterol was classified as <200 mg/dL (ideal) and 200–239, or treated as <200 mg/dL (intermediate) and ≥240 mg/dL (poor). Blood pressure was categorized into SBP < 120 mm Hg and DBP < 80 mm Hg (ideal), SBP 120–139 mm Hg or DBP 80–89 mm Hg, or treated as a normal level (intermediate), as well as SBP ≥ 140 mm Hg or DBP ≥ 90 mm Hg. For fasting glucose, the ideal, intermediate, and poor status was defined as <100 mg/dL, 100–125 mg/dL or treated as normal, and ≥126 mg/dL.

Participants were given 0 (poor), 1 (intermediate), or 2 (ideal) points for each component based on their status for each of the CVH metrics. The cardiovascular score was calculated as a summary score of the seven components and further categorized as tertiles, with scores ranging from 0–6, 7–8, and 9–12.

### 2.3. Proteomic Assessment

Measurement of plasma proteins was conducted using the 1.3k SOMAscan assay at the Trans-NIH Center for Human Immunology, Inflammation, and Autoimmunity (CHI), the National Institute of Allergy and Infectious Disease, and the National Institutes of Health (Bethesda, MD, USA), following protocols previously described [21]. Overnight fasted plasma was collected and stored at −80 °C. Stored plasma from baseline samples (1998–2000) were used to assess 1322 SOMAmer Reagents. These SOMAmer Reagents included 12 hybridization controls and 4 viral proteins (HPV type 16, HPV type 18, isolate BEN, isolate LW123). Further, five SOMAmer Reagents were flagged for non-specific binding (P05186; alkaline phosphatase, P09871; complement component 1, Q14126; desmoglein 2, Q93038; tumor necrosis factor receptor superfamily member 25, Q9NQC3; reticulon-4). The hybridization controls, viral proteins, and flagged SOMAmer Reagents were removed, resulting in a total of 1301 SOMAmer Reagents that were used in the final analysis. Some of the SOMAmer Reagents are designed to capture multiplex proteins with two or more unique proteins. Thus, the 1301 SOMAmer Reagents measure 1297 unique proteins (or Uniprot IDs). For the purposes of this project, “protein” will refer to an individual SOMAmer Reagent.

The protocol for data processing, including normalization for hybridization, control normalization, median signal normalization, and calibration normalization, has been detailed in previous publication [22,23]. An abundance of plasma proteins are evaluated as relative abundances of SOMAmer Reagents. The data are reported as relative fluorescence units (RFUs) that are directly proportional to the reported relative abundance of SOMAmer Reagents.

### 2.4. Statistic Analysis

All statistical analyses were performed using R version 4.2.1. Differences in baseline characteristics by tertiles of CVH score were assessed by one-way ANOVA for continuous variables and chi-square test for categorical variables. To identify the proteins associated with CVH, protein RFU abundances were natural-log transformed and outliers outside of four standard deviations were removed from the analyses. Associations were tested using linear regression models, using CVH as the main dependent variable. The models were adjusted for age, sex, study site (Greve in Chianti or Bagno a Ripoli), and 24-h creatinine clearance. To understand which component of CVH, if any, was driving the association between proteins and CVH, associations with the 92 CVH-association proteins with individual components of CVH (smoking, diet, physical activity, fasting glucose, blood pressure, total cholesterol, and BMI) were tested using linear regression models, adjusting for the same covariates. Each CVH component was analyzed using the three-category variable used to construct CVH as a continuous variable. Specifically, the categories poor, intermediate, and ideal was coded as 0, 1, and 2 and treated as a linear independent variable. Therefore, the beta estimates from the regression model indicates the linear association of proteins with better CVH components. For example, a positive association between a plasma protein with a BMI component would indicate a higher protein abundance with lower BMI, and conversely a negative association would reflect a lower protein abundance with lower BMI. For all proteomic analyses, correction for multiple comparisons was applied using a Benjamini-Hochberg false discovery rate (FDR), and significance was considered at an adjusted *p* ≤ 0.05.

The association between CVH and all-cause mortality was evaluated using cox proportional hazards regression, using *coxph* function in survival package (version 3.4). The mediating effect of proteins on the association between CVH and all-cause mortality was tested using mediation analysis implemented via the *regmedint* package (version 1.0). The natural indirect effect estimated reflects the effect of CVH on all-cause mortality through the mediator, plasma protein. The statistical significance of this mediated effect was estimated for the proteins that were identified through the previous proteomic analysis of CVH.

## 3. Results

### 3.1. Association of Plasma Proteins with Cardiovascular Health

Of the 1155 participants over 65 years of age at baseline, 703 participants had complete data on plasma proteins, mortality, and CVH. Participants included in these analyses had a lower mean age, percentage of women, CVH score, and physical activity level compared to those without complete data. Conversely, those with complete data had higher creatinine clearance and total cholesterol than those without complete data (Supplementary Table S1). In the analytic sample, there were no significant differences in distribution by age, sex, study site, and creatinine clearance by tertiles of CVH (Table 1).

**Figure 1:**
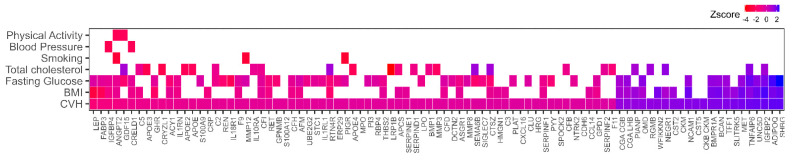
Heatmap displaying the associations between plasma proteins with cardiovascular health (CVH) and individual components of CVH: smoking, physical activity, smoking, blood pressure, total cholesterol, fasting glucose, and body mass index (BMI). None of the CVH associated proteins were associated with dietary intake component.

Consistent with our prior report [24], in the analytic sample used in this study, higher CVH was inversely associated with mortality in the InCHIANTI participants (β = −0.11 (HR = 0.89), *p* = 1.17 × 10^−5^). To identify pathways that mediate this association, we examined the association between CVH score with 1301 plasma protein levels. There were 92 proteins (22 positively, 70 negatively) that were associated with CVH score (Figure 1, Supplementary Table S2). The proteins most strongly negatively associated with CVH were leptin (LEP), fatty acid binding protein 3 (FABP3), Angiopoietin-2 (ANGPT2), and growth differential factor 15 (GDF15). Functional analysis of the 92 significant proteins showed significant enrichment of the “complement and coagulation cascades” KEGG pathway with 14 represented proteins: complement component 2, 3, and 5 (C2, C3, C5); complement factors B, D, H, and I (CFB, CFD, CFH, CFI); clusterin (CLU); coagulation factor IX and XI (F9, F11); tissue plasminogen activator (PLAT); serpin family D member 1 (SERPIND1); plasminogen Activator Inhibitor 1 (SERPINE1); and alpha-2-antiplasmin (SERPINF2).

Associations with individual CVH score components indicate that of the 92 proteins, 62 proteins (67.4%) were associated with BMI, 57 proteins (62%) with fasting glucose, 41 proteins (44.6%) with total cholesterol, 28 proteins (30.4%) with blood pressure, 21 proteins (22.8%) with physical activity, 13 proteins (14.1%) with smoking, and 3 proteins (3.3%) with diet (Figure 1; Supplementary Table S3). These findings suggest BMI and fasting glucose were the main drivers of proteomic differences observed with CVH.

### 3.2. Plasma Proteins Mediate the Association between CVH and Mortality

**Figure 2:**
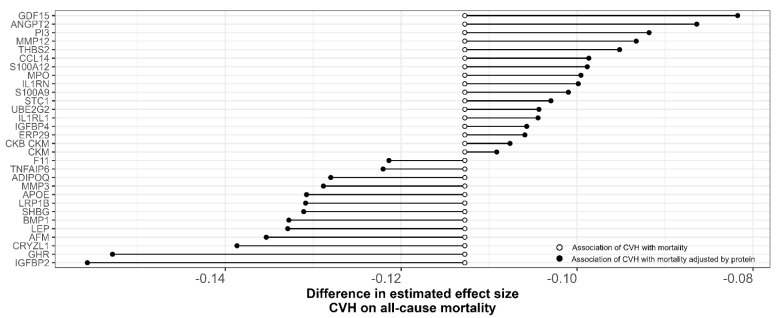
The mediating effect of individual plasma protein on the relationship between CVH and all-cause mortality. Higher CVH was negatively associated with all-cause mortality (white circle). Inclusion of individual plasma proteins significantly changed the magnitude of association (black circle), reflecting the mediation effect. Of the 92 CVH associated proteins, 33 proteins significantly mediated the relationship between CVH and all-cause mortality.

A mediation analyses was conducted to determine whether the 92 identified proteins mediated the association of CVH with mortality. We found that 33 proteins significantly mediated the association, with percent mediation ranging from 5 to 30% (Figure 2; Supplementary Table S4). The strongest mediation was observed for GDF15, insulin-like growth factor 2 (IGFBP2), and growth hormone receptor (GHR).

## 4. Discussion

In this study we examined the plasma proteomic profiles associated with CVH, operationalized using the life simple 7 metric, to uncover the possible mechanisms underlying the protective effect of CVH on mortality. We found that ideal CVH had a significant impact on protein homeostasis that was reflected by the differential abundances of many proteins spanning different biological domains. Specifically, we identified a set of 92 proteins associated with CVH, and 33 of these proteins significantly mediated the association between CVH and mortality. The proteins identified represented different biological pathways and health states including complement and coagulation cascade, growth hormone (GH) and insulin-like growth factor (IGF) pathway, obesity-related proteins, and aging proteins. These results provide insight into the biological processes that are influenced from having better CVH and consequently promote longevity.

The protein with the greatest mediating effect between CVH and mortality was GDF-15, which is known as one of the most significant age-associated proteins [21,25]. In this study, GDF15 was found to be negatively correlated with CVH, and positively associated with older age. In fact, other proteins that mediate the relationship between CVH and mortality are age-associated proteins, including MMP12, STC1, CCL14, PI3, and THBS2 [21,25]. For many of the proteins associated with both age and CVH, the direction of association between CVH and protein abundance is in the opposite direction to the association between age and protein abundance. This suggests that the proteome of individuals with favorable CVH reflects a “younger” proteomic profile. In fact, as we describe below, the proteins associated with CVH and mortality represent key pathways in the aging process.

Many proteins in the complement (C2, C3, C5, CFB, CFD, CFH, CFI, CLU) and coagulation cascade (PLAT, SERPIND1, SERPINE1, SERPINF2, F11, F9) were negatively correlated with CVH. The complement and coagulation cascade are pathways that are often simultaneously activated in response to an infection or tissue damage, which in turn activate inflammation [26]. The proteins representing the complement pathways that were underrepresented with better CVH included many of the major complement and complement factor proteins involved in the cascade including C2, which forms the serine protease C3 convertase when binding with C4b. The C3 convertase in turn cleaves C3 to C3a and C3b. The product C3b then combines with C4 the major complement proteins C3, which are cleaved into C3a, and which in turn form C5 convertase that cleaves C5. Several proteins in the intrinsic coagulation cascade and fibrinolysis pathway were underrepresented with better CVH. These included the two fibrinogen factors XI (F11) and IX (F9) as major serine proteases in the intrinsic coagulation cascade. The proteins in the fibrinolysis pathway included a thrombin inhibitor, SERPIND1, PLAT, that catalyzes the conversion of plasminogen to plasmin, as well as its inhibitor, SERPINE1, and a plasmin inhibitor, SERPINEF2. Activation of the complement and coagulation cascade in pathogenesis of various diseases including cardiovascular disease [27] and Alzheimer’s disease [28,29] were documented. Our data support that these pathways are suppressed in individuals with higher CVH.

Out of the proteins in the complement coagulation pathway, F11 was the only protein that mediated the relationship between CVH and mortality. As described previously, F11 is a coagulation enzyme that is activated by serine protease XIIa in the intrinsic coagulation cascade [30]. Interestingly, patients with rare inherited coagulation factor deficiencies with middle to severe reduction in F11 activity had a lower incidence of cardiovascular events including myocardial infarction, stroke and transient ischemic attach, and venous thromboembolism [31]. In observational studies, elevated circulating F11 has been associated with an increased risk of thrombosis and cerebrovascular events, as well as recurrent venous thrombosis [32,33]. In addition to cardiovascular disease, plasma F11 was one of the proteins that discriminated between frail and non-frail participants in a study of older Italians [34]. These studies, taken together, support F11 as an important determinant of health span. Consistent with these observations, our study supports the idea that improved CVH may reduce the risk of mortality through suppression of F11.

Three proteins in the growth hormone (GH) and insulin-like growth factor (IGF) pathway, GH receptor (GHR), IGF binding protein 2 (IGFBP2), and IGFBP4, significantly mediated the association between CVH and mortality. The GH/IGF pathway has a primary role in growth, but also influences many physiological processes, including glucose and lipid homeostasis [35]. In human aging, the activity of the GH/IGF pathway decline in older age leads to the hypothesis that augmenting somatotropic hormones may a have beneficial effect on health [36]. However, human GH replacement was shown not to be an effective anti-aging strategy through trials that had modest effects on body composition with unfavorable side effects including soft tissue edema, carpal tunnel syndrome, and increased risk of developing diabetes and impaired fasting glucose [37]. In fact, there is mounting evidence indicating that suppression of the GH/IGF pathway is beneficial to health and lifespan. In animal models with reduced GH/IGF, pathway activity has been associated with increased longevity and health span. In studies of human centenarians and nonagenarians, there was an inverse relationship between IGF-1 and IGF1/IGFBP3 with survival supporting the benefits of GH/IGF suppression for human longevity [36,38]. Further, there is evidence from epidemiological studies of the inverse relationship between the GH/IGF pathway activation and age-related diseases such as cardiovascular disease [39] and a decline in cognitive function [40]. However, not all studies reported significant [41,42] or protective associations [43,44]. In the present study, there was no significant association between CVH and IGF-1 abundance. However, IGFBP2 was found in higher abundance, while IGFBP4 and GHR were found in lower abundance with better CVH. Growth hormone inhibits the expression of IGFBP2, while stimulating the expression of IGFBP4 [45]. Therefore, the pattern of association observed in the current study supports the downregulation of the GH/IGF pathway with improved CVH, which in turns has beneficial effects on mortality.

In our analysis, leptin was negatively associated with CVH; in particular, leptin was significantly associated with the BMI component of CVH, where lower leptin was observed with lower BMI. Leptin, a 167-amino-acid product of the human leptin gene, is secreted by white adipose tissue, and its levels are positively correlated with body fat [19]. This is consistent with what was observed in this study. Other studies support our finding; for example, among participants 60 years and older belonging to the National Health and Nutrition Examination Survey III (1988–1994), cardiovascular mortality was in the highest tertile of leptin levels for men. In sensitivity analysis, results were particularly stronger for men with central obesity and higher body fat [46]. A 2017 meta-analysis that included 13 case-control and cohort studies concluded that while high leptin levels were associated with coronary heart disease mortality in simple models, further adjustment for other established heart disease risk factors resulted in the loss of statistical significance [47]. Our study supports these previous findings and suggest that proteomic profile consistent with lower BMI may be one of the mechanisms linking CVH and mortality.

This study is the first to evaluate the proteomic profile of CVH and its relationship with mortality. Using data from a well-characterized cohort, we identified candidate proteomic biomarkers that may mediate the relationship between CVH and mortality. However, this study has several limitations. First, the study was conducted in a population-based study of older Italian adults, thus the generalizability of the results may be limited to samples with similar demographic and geographical characteristics. Second, while the proteomic assessment included 1300 proteins, there are circulating proteins that were not assessed in this study. Further replication of our finding using proteomic assessment technologies other than the aptamer-based methods will be important to validate our findings. Finally, the proteomic assessment method measured relative abundance, and other post-translational modification such as phosphorylation and acetylation may be important in assessing CVH health. Based on evidence on the role of sleep in chronic disease risk, the American Heart Association recently updated the metric to include sleep as part of the health behavior component (in addition to the existing components) [48]. We are currently in the process of updating the CVH metric to reflect this change, and next steps will include repeating these analyses to determine whether the proteomic profile will differ with the updated Life’s Essential 8 to examine whether the updated metric is associated with mortality and changes in the proteomic mediators.

In summary, our proteomic analysis of CVH identified differential abundance of proteins that point towards multiple molecular pathways including inflammation, complement and coagulation, and GH/IGF pathway that may be mediating the protective effect of CVH on mortality. These proteins potentially have clinical utility as biomarkers to monitor overall cardiovascular health or to be investigated as potential therapeutic targets.

## Figures and Tables

**Table 1:** Characteristics of InCHIANTI participants by tertile of CVH score.

	All	Tertile 1 Score 0–6	Tertile 2 Score 7–8	Tertile 3 Score 9–12	*p*
n	703	191	275	237	
Age (years)	73.3 (6.24)	73.6 (6.35)	73.5 (6.20)	72.9 (6.21)	0.479
Female (%)	54.2	56.0	56.4	50.2	0.318
Bagno a Ripoli (%)	52.5	52.4	51.6	53.6	0.907
Creatinine clearance (mL/minute)	77.8 (24.9)	75.9 (24.2)	76.9 (23.5)	80.5 (26.8)	0.123
Cardiovascular Health (CVH score)	7.61 (1.90)	5.25 (1.04)	7.48 (0.50)	9.67 (0.85)	<0.001
BMI (kg/m^2^)	27.5 (4.03)	29.6 (4.27)	27.7 (3.94)	25.6 (2.93)	<0.001
Fasting Glucose (mg/dL)	95.4 (24.3)	109.3 (32.2)	92.8 (21.6)	87.3 (12.3)	<0.001
Total cholesterol (mg/dL)	219.9 (38.3)	234.0 (38.38)	223.9 (35.2)	204.0 (36.0)	<0.001
Mediterranean diet score	4.52 (1.63)	3.79 (1.58)	4.47 (1.56)	5.17 (1.48)	<0.001
Systolic blood pressure (mmHg)	150.0 (19.3)	154.3 (16.0)	152.1 (19.2)	144.0 (20.4)	<0.001
Diastolic blood pressure (mmHg)	84.0 (8.45)	85.9 (7.80)	84.4 (8.15)	82.0 (8.90)	<0.001
Physical activity (%)					<0.001
Low	15.2	32.5	14.2	2.5	
Medium	45.1	53.4	51.6	30.8	
High	39.7	14.1	34.2	66.7	
Smoking (%)					<0.001
Never smoked	57.2	42.4	57.1	69.2	
Former smoker	28.2	30.4	29.5	24.9	
Current smoker	14.7	27.2	13.5	5.9	

Values represent mean (SD), or n (%).

## Data Availability

Data from InChianti study is available through collaboration through submission of a proposal at https://www.nia.nih.gov/inchianti-study. Please contact the corresponding author for further information.

## Supplementary Materials

The following supporting information can be downloaded at: https://www.mdpi.com/article/10.3390/nu15030781/s1, Table S1: Demographic and Clinical characteristic of InCHIANTI participants with and without proteomic data; Table S2: Association of plasma proteins with cardiovascular health; Table S3: Association of plasma proteins with cardiovascular health components; Table S4: Indirect effect of cardiovascular health associated proteins on all-cause mortality.
